# Supplementary material for: Flickering candle flames and their collective behavior
Source: Sci Rep. 2020 Dec 4;10:21305. doi: 10.1038/s41598-020-78229-x (PMC7719181; doi:10.1038/s41598-020-78229-x)
Supplement: Supplementary file 1 — Supplementary Information. [file 41598_2020_78229_MOESM1_ESM.pdf]

Flickering candle flames and their collective behavior

Attila Gergely[1], Bulcsú Sándor[1], Csaba Paizs[2], Robert Tötös[2], and Zoltán Néda[1,*]

[1]Department of Physics, Babeş-Bolyai University, Kogălniceanu street nr. 1, RO-400084 Cluj-Napoca, Romania
[2]Biocatalysis and Biotransformation Research Centre, Faculty of Chemistry and Chemical Engineering, Babeş-Bolyai University, Arany János street nr. 11, RO-400029, Cluj-Napoca, Romania
[*]zneda@phys.ubbcluj.ro

SUPPLEMENTARY MATERIALS

A: Halogen bulb - candle flame interaction
B: Synchronization order parameter
C: Coupling mechanism for the collective behavior

Supplementary Materials

A. Halogen bulb - candle flame interaction

In order to investigate the role of thermal radiation in the formation of the collective behavior of the flames we used a pulsating radiation source with the same radiation properties as the candle flame. This was provided by a halogen bulb of 50W which had the same spectra as the one of the candles flame. Comparison between the radiation spectrum of the bulb and the candle flame is given in Figure 1. We learn from here that the relative intensity distributions of the two spectra are similar in shape. On Figure 2 we show the circuit diagram used to modulate the current of a halogen bulb so that oscillations of any given frequency can be achieved in the intensity of the emitted thermal radiation.

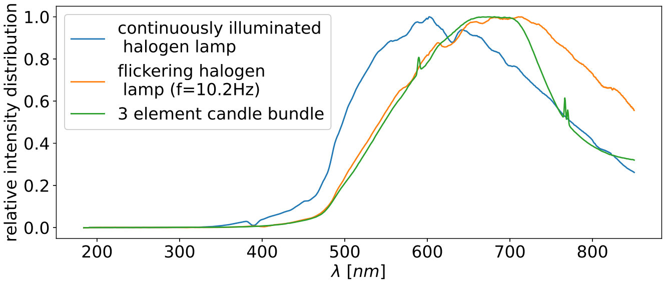

Supplementary Figure 1. The figure above shows the emission spectra of the halogen bulb, the flickering halogen bulb, and the candle bundle flame. It can be seen that the thermal emission of the flickering bulb is almost identical to the emission of the candle bundle.

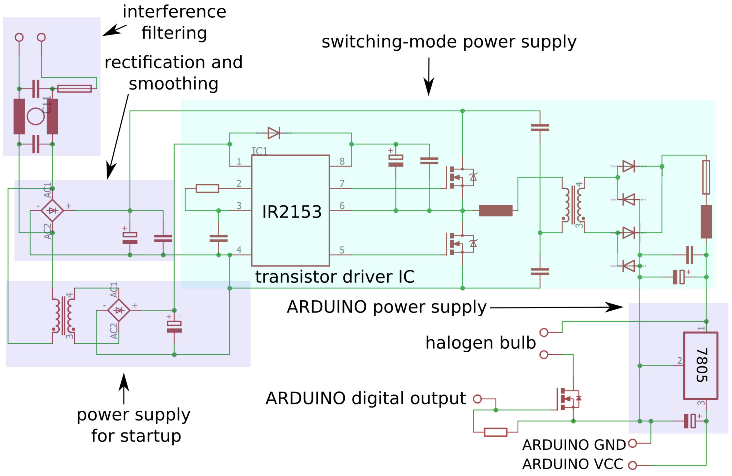

Supplementary Figure 2. A current regulator controlled by a pulse-width modulation signal. The circuit provides power to the halogen bulb, while the control signal is provided by an Arduino. The halogen light bulb driven by the above circuit generates thermal radiation with oscillating intensity at a fixed frequency.

B. Synchronization order parameter

The z synchronization parameter is computed using the method presented in[1]. For explaining the method let us consider two time series shown in Figure 3.

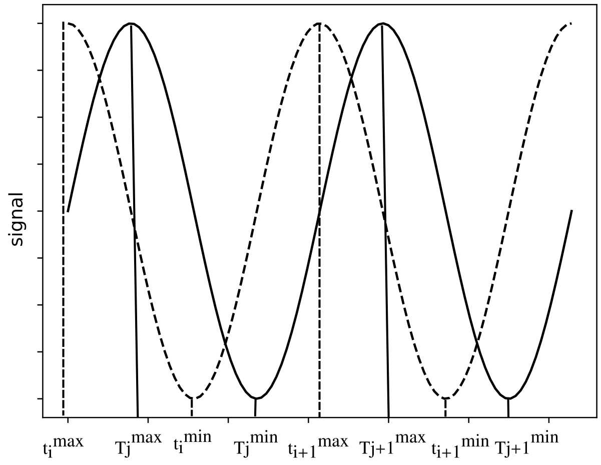

Supplementary Figure 3. Two periodic time series used to describe the notations in computing the z synchronization parameter.

We denote by t_i^{min} and t_i^{max} the i-th local minimum and maximum of the time series represented by the dashed line. Similarly, T_j^{min} and T_j^{max} denotes the j-th local minimum and maximum of the time series indicated by the solid line. With the help of two additional quantities, t_1 and t_2 are defined

$$t_1 = \langle \min_{\{i\}} \{|t_i^{max} - T_j^{max}|\} \rangle_j, \tag{S1}$$

$$t_2 = \langle \min_{\{i\}} \{|t_i^{min} - T_j^{max}|\} \rangle_j, \tag{S2}$$

the synchronization parameter is obtained as:

$$z = \frac{t_2 - t_1}{t_2 + t_1} \tag{S3}$$

C. Coupling mechanism for the collective behavior

We propose a coupling mechanism for the dynamical equations proposed in (**??**) through the air movement induced by the candle flickering. The main hypothesis is that any change in one of the flames size will induce an air-flow to the nearby flames. A simple model based on the expansion of an ideal gas is used. We approximate the material inside the flame with an ideal gas. When the temperature changes inside this the volume of the gas changes. For simplicity we assume an isobar expansion. If we denote the pressure by p, the thermodynamic temperature inside the flame as T, the gas quantity inside the flame as ν, the ideal gas constant by R, and approximate the flame with a sphere of radius r (for the notations please follow Figure 4), from the thermal equation of state for ideal gases one gets:

$$dT = \frac{4\pi p}{\nu R} r^2 \, dr = C r^2 \, dr \tag{S4}$$

Here C denotes a constant characteristic for the considered flame. Assuming now that the change of the size in the flame induces a displacement in the surrounding air which propagates at larger distances, we can write up the displacement at a distance x:

$$dx = \frac{r^2}{x^2} dr = \frac{1}{C} \frac{dT}{x^2} \tag{S5}$$

The amount of extra oxygen reaching the nearby flame at distance x can be thus estimated as

$$dn \propto |dx| \rightarrow \frac{dn}{dt} = \frac{\gamma}{x^2} \left| \frac{dT}{dt} \right|, \tag{S6}$$

where γ is yet another constant. The modulus is due to the fact that displacements in the size of the flame in both directions will induce extra oxygen concentration in the nearby flames.

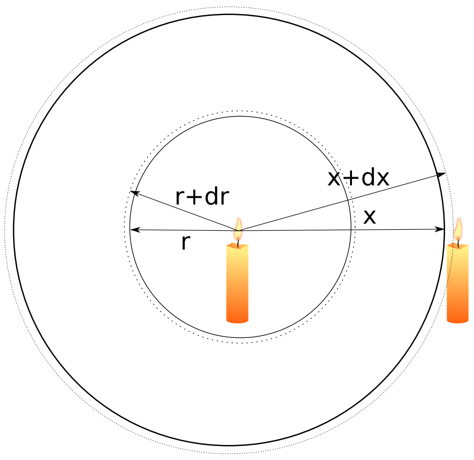

Supplementary Figure 4. Sketch and notations for the coupling mechanism considered through the air displacements arround the flames.

References

1. Boda, S., Néda, Z., Tyukodi, B. & Tunyagi, A. The rhythm of coupled metronomes. *The Eur. Phys. J. B* **86**, DOI: 10.1140/epjb/e2013-31065-9 (2012).
